# The effects of temporal pressure on obstacle negotiation and gaze behaviour in young adults with simulated vision loss

**DOI:** 10.1038/s41598-019-51926-y

**Published:** 2019-10-28

**Authors:** Tjerk Zult, Jonathan Allsop, Matthew A. Timmis, Shahina Pardhan

**Affiliations:** 1 Vision and Eye Research Institute, School of Medicine, Faculty of Health, Education, Medicine, and Social Care, Anglia Ruskin University, Cambridge, United Kingdom; 2 Central Flying School, Royal Air Force College, College Cranwell, Sleaford, United Kingdom; 3 Cambridge Centre for Sport and Exercise Science, Anglia Ruskin University, Cambridge, United Kingdom

**Keywords:** Skeleton, Vision disorders, Risk factors

## Abstract

Individuals with vision loss adapt their locomotion and gaze behaviour to safely negotiate objects in temporally unconstrained situations. However, everyday activities are often performed under time-pressure. We investigated the effects of blur on anxiety, movement kinematics and gaze behaviour during the negotiation of a floor-based obstacle under three amounts of pressure: 1) no-pressure; 2) tonal-pressure: an intermittent tone was played at a constant frequency; 3) tonal + time pressure: the intermittent tone increased in frequency and participants had to walk 20% faster to reach the end of the lab. Irrespective of the amount of pressure, the blurred vs. normal vision group reported 32% more anxiety, lifted the lead foot 43% higher and 10% slower over the obstacle, and looked 6% longer and 6% more frequently ahead of the obstacle. In the tonal + time pressure vs. no-pressure condition, both groups were more anxious, showed adaptations in movement kinematics related to walking faster, and adopted a ‘checking strategy’ by shortening their fixation durations at the obstacle. These results show that irrespective of temporal pressure, the blurred vision group remained more cautious as to how the lead foot negotiated the obstacle, in order to reduce the chance of tripping during crossing.

## Introduction

The coupling between the visual and locomotor system allows humans to safely negotiate a hazard when it appears in their travel path. This may result in either navigating around the hazard/object or stepping over it. When walking up to and safely stepping over an object, individuals with normal vision rely on a combination of central and peripheral vision to adapt their walking gait^1,2^. Individuals typically look several steps ahead^3–5^ fixating on the obstacle and task-relevant areas to plan their foot placement^4^. In addition, peripheral visual information is utilised for the online “fine tuning” of gait by providing exproprioceptive information (i.e., position of the lower limbs relative to the environment)^2,6,7^. Individuals with refractive error or cataract adapt their locomotion^8–12^ and gaze^13,14^ to safely navigate through the environment. Importantly, in all the aforementioned literature, gaze behaviour and adaptive gait were assessed when individuals were temporally unconstrained. However, everyday activities are often performed under some element of time-pressure. For example, a ringing telephone, which is positioned on the other side of the room, presents a time constraint situation whereby they are required to walk across the room and answer the phone before it stops ringing. To date, it remains unclear how the addition of a time-pressure situation impacts adaptive gait and gaze behaviour in individuals with visual impairment.

It is possible that in a time constrained situation, adaptive gait is modified due to an increase in anxiety. Time-pressure situations are known to induce anxiety during surgical practice^15,16^ and impair surgical performance^16,17^. Anxiety influences motor performance as well as the perception of environmental characteristics and the selection of action possibilities^18,19^. In sports, anxious people are more easily distracted by task-irrelevant information and they adopt an ineffective visual search strategy (i.e., fixations of shorter duration on task-relevant areas)^20^ – behaviours that strongly correlate with poorer execution of subsequent motor actions^21–23^.

The effect of anxiety on visual search behaviour has not been extensively studied in everyday tasks such as locomotion, but recent literature has proposed that fall-related anxiety could impair visual search behaviour when walking through a complex environment^24,25^. Indeed, older adults with high compared to low self-reported anxiety fixated earlier and for longer duration on the target area for foot placement and fixated less often and for shorter duration at subsequent constraints in the travel path^26^. Interestingly, older adults with high self-reported anxiety also showed less accurate foot placement in the target area even though they spent longer time fixating on this area^26^. According to the integrated model of perceptual-motor performance^18,19^, anxious performers might adopt an inward focus of attention in which perceptual-motor tasks that are normally performed automatically (such as locomotion) now require conscious processing. Consequently, the automaticity of the motor task is disrupted which affects the fluidity of the movement and increases the error rate^27^.

A reduction in the automaticity of locomotion has been observed with increased levels of anxiety^28–32^, and is more evident in fallers than non-fallers^33,34^. Allocating conscious attention to locomotion is cognitively demanding and reduces the attentional resources available for executing other task-relevant processes^28^. Consequently, task-relevant processes other than locomotion, such as the awareness of hazards in the environment, become less of a priority^30,32,35,36^. To illustrate, individuals with a conscious gait control, whether induced by anxiety or not, were less aware of their environment^30,35^ and fixated for shorter durations on the task-relevant areas in their travel path^36^. A recent study showed that an anxiety-induced internal focus of attention resulted in rapid initial fixations toward the more proximal areas of the travel path, suggesting hypervigilance toward immediate threats in the environment at the expanse of planning future stepping actions^32^. This interpretation is in line with the idea that anxiety increases the influence of the stimulus-driven system (i.e., immediate threats) at the expense of the goal-directed system (i.e., scanning the entire travel path to plan future stepping actions)^37^.

Less is known about the influence of anxiety on the movement kinematics of locomotion. Studies on obstacle negotiation showed that increased levels of anxiety resulted in a safer obstacle crossing strategy (i.e., higher clearance of the lead foot and a slower clearance of the lead and trail foot)^38^ and a lower number of obstacle contacts^39^. Thus, higher levels of anxiety might actually decrease the chance of tripping on an obstacle. These studies^38,39^ were both performed in individuals with normal vision so it remains unclear how individuals with visual impairment would behave in similar anxiety inducing situations.

Anxiety levels in no-pressure situations can be twice as high in individuals with vision loss compared to controls^40,41^. In addition, level walking with vision loss requires more mental effort^42,43^ and increases the participation of brain areas involved in sensory processing, motor planning, and motor execution^44^. The involvement of these brain areas is further enhanced when negotiating an obstacle^45^ and the cognitive demand is supposedly even higher in individuals with vision loss. Thereby, it is postulated that vision loss will result in a more conscious control of locomotion^46^, further increasing the cognitive load with less attentional resources available for other task-relevant processes.

The higher anxiety levels in individuals with vision loss^40,41^, accompanied by a more conscious control of locomotion^46^, likely increase the cognitive load and require adaptations in locomotion^8–12^ and gaze behaviour^13,14^ to safely navigate through the environment. To illustrate, individuals with (simulated) cataract walked slower^8,10–14^, adopted a more cautious gait (i.e., a higher and slower clearance of the lead foot over the obstacle^9^ and an increased toe clearance when ascending a step^8^). They also looked down more frequently and for longer when walking through a complex environment^14^. These behaviours were also observed when individuals with normal vision performed such tasks under increased levels of anxiety, whether coinciding or not with a more conscious control of gait^26,32,38,39^. However, it is currently unknown how individuals with visual impairment will behave in anxiety inducing situations (simulated through increased time pressure) and whether this behaviour is different from individuals with normal vision.

The present study examines the effects of time-pressure and simulated blur on movement kinematics and gaze behaviour during the performance of an obstacle crossing task. The performance of a time constrained task is known to increase anxiety levels. It is expected that in the no pressure (habitual) condition, individuals with simulated loss of vision will negotiate the obstacle more cautiously (i.e., foot placement further away from the obstacle, higher and slower obstacle crossing with both feet, and longer single support times), and will fixate longer and more frequently toward more proximal areas in the travel path at the expense of looking at more distal areas such as the obstacle. Under time-pressure, the expected increase in anxiety will be higher for individuals with simulated loss of vision which will lead to a more cautious obstacle crossing strategy with more frequent but shorter fixations at more proximal areas in the travel path compared to the normal vision group.

## Results

The main aim of the study was to determine whether individuals with and without vision loss become more anxious, and if they alter movement kinematics and gaze behaviour when negotiating an obstacle under increasing time pressure. The participants were randomly assigned to a normal vision group (*n* = 14) and blurred vision group (*n* = 14). Blurred vision was simulated using full aperture trial case lenses. The minimal visual acuity that participants were impaired to was 0.80 logMAR. Group characteristics can be found in Table 1. Participants were instructed to negotiate a 10 cm high obstacle in the middle of their travel path under three amounts of pressure: 1) no pressure; 2) tonal pressure: an intermittent tone was played at a constant frequency; 3) tonal + time pressure: the intermittent tone increased in frequency and participants had to walk 20% faster to reach the end of the lab before the tone extinguished. The tonal pressure condition was added to confirm that the effects in the tonal + time-pressure condition were evoked by the temporal (time) component and not the tonal (sound) component of the task.

**Table 1:** Baseline characteristics of the participants (mean ± SD).

	Blurred vision group (n = 14)	Normal vision group (n = 14)
Age (*years*)	32 (5)	27 (4)
Sex		
Male	7	9
Female	7	5
Mass (*kg*)	69 (12)	74 (19)
Height (*cm*)	172 (10)	172 (12)
Visual acuity (*logMAR*)	0.95 (0.07)	−0.16 (0.09)

Of note, visual acuity in the blurred vision group reflects their acuity with the blurred lenses.

Participants started the experiment with nine baseline walking trials (no obstacle present) at their comfortable walking speed (i.e., to get familiar with the task) followed by the obstacle negotiation trials under three pressure condition. Each condition consisted of four trials of which three were obstacle trials and one was a level walking trial. Prior to the start of each trial, the participants’ view of the travel path was obstructed and participants were unaware whether the trial was an obstacle or level walking trial. Participants were aware of which pressure condition they would face. Practice trials were not allowed for any condition. Participants performed 21 trials in total. Self-reported anxiety and perceived temporal demand were examined after each condition (i.e. after finishing the block of trials in that condition) and movement kinematics and gaze behaviour were simultaneously recorded during each trial.

To date, adaptive gait and gaze behaviour are only assessed when individuals were temporally unconstrained. However, everyday activities such as stepping up the pavement after crossing a road are often performed under an element of time-pressure (e.g., a traffic signal that gives pedestrians limited time to cross). The performance of a time constrained task is known to increase anxiety levels^15,16^ and anxiety levels in no-pressure situations can be twice as high in individuals with vision loss compared to those with normal vision^40,41^. Individuals with normal vision adopted a more cautious obstacle crossing strategy in anxiety inducing situations (i.e., higher clearance of the lead foot and a slower clearance of the lead and trail foot)^38^, contacted the obstacle less often^39^, and looked down more frequently and for longer when performing an obstacle course^26,32,38,39^. Similar adaptations in adaptive gait^8–12^ and gaze behaviour^13,14^ were observed in individuals with (simulated) cataract/blur when navigating through the environment without time pressure. However, it is currently unknown how individuals with visual impairment will behave in anxiety inducing situations (simulated through increased time pressure) and whether this behaviour is different from individuals with normal vision. It is expected that in the no pressure (habitual) condition, individuals with simulated blur will adopt a more cautious obstacle crossing strategy (i.e., foot placement further away from the obstacle, higher and slower obstacle crossing with both feet, and longer single support times), and will fixate longer and more frequently toward more proximal areas in the travel path at the expense of looking at more distal areas such as the obstacle. Under time-pressure, the expected increase in anxiety will be higher for individuals with simulated blur which will lead to a more cautious obstacle crossing strategy with more frequent but shorter fixations at more proximal areas in the travel path compared to the normal vision group.

### Task performance times

**Figure 1:**
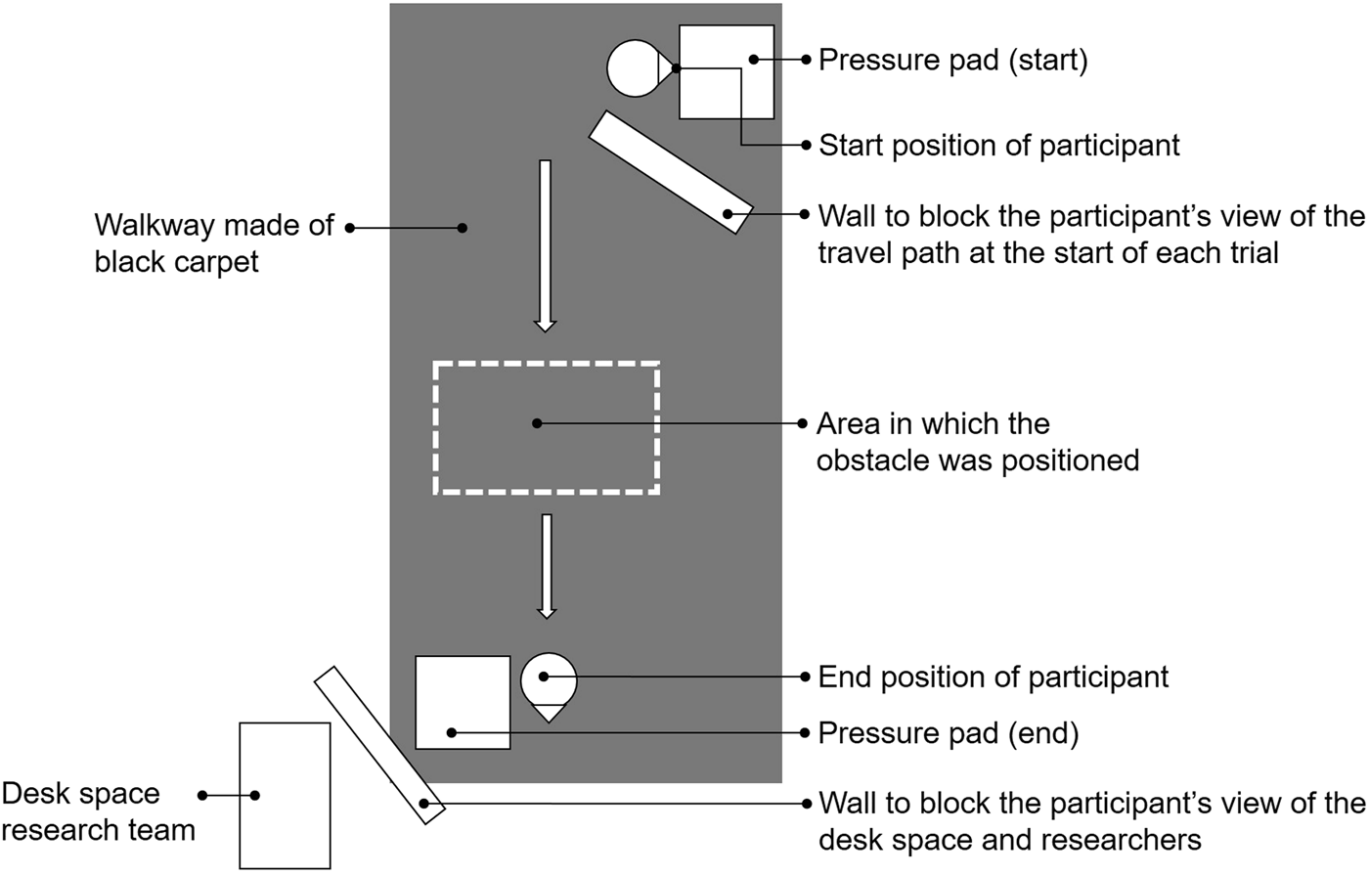
Schematic representation of the experimental setup.

Trial times were determined from the instant that the right hand was removed from the pressure pad at the start of the trial until the right hand was placed on the pressure pad at the end of the walkway, denoting the end of the trial (Fig. 1). In the tonal + time pressure condition, 5 participants in the normal vision group and 3 participants in the blurred vision group failed to reach the end of the walkway before the tone ceased (16 out of 112 trials, 14%). In these 16 trials, participants ended the trials 0.20 ± 0.24 seconds after the tone ceased. The statistical analysis was performed with and without these 16 trials and the outcomes were not affected. The reported data are for the full dataset. None of the participants contacted the obstacle.

**Figure 2:**
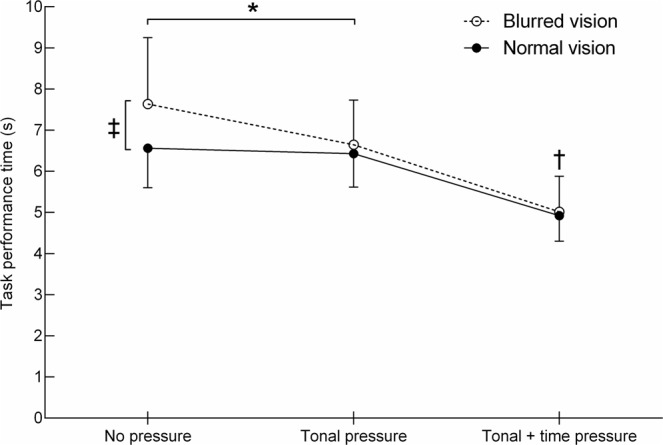
Task performance times of the normal vision and blurred vision group in the three different conditions (mean ± SD). ^‡^Between group difference in the no pressure condition (*p* < 0.05). ^†^Condition effect for both groups in the tonal + time pressure condition vs. tonal pressure and no pressure condition (*p* < 0.05). *Group by condition interaction between the tonal pressure and no pressure condition (*p* < 0.05).

Figure 2 and Table 2 illustrate that there was no main effect of group for task performance time (*p* = 0.174). There was a condition effect (*F*_2,52_ = 72.6, *p* < 0.001) and group by condition interaction (*F*_2,52_ = 4.3, *p* = 0.019). Post hoc testing for the interaction effect revealed that the blurred vision group compared to the normal vision group performed the task 16% slower in the no pressure condition (*p* = 0.041, *d* = 0.75), but similarly in the tonal pressure and tonal + time pressure conditions (*p* ≥ 0.555). On comparing between the conditions for each group, the blurred vision group performed the task 11% faster in the tonal pressure vs. no pressure condition (*p* = 0.001, *d* = 0.73), while performance times in the normal vision group were not significantly different between these two conditions (*p* = 0.626). Both groups showed faster performance times in the tonal + time pressure condition compared to the no pressure and tonal pressure condition (all *p* ≤ 0.001).

**Table 2:** Movement kinematics of the normal vision and blurred vision group in the three different conditions (mean ± SD).

Variables	Normal vision group (n = 14)			Blurred vision group (n = 14)			ANOVA
	No pressure	Tonal pressure	Tonal + time pressure	No pressure	Tonal pressure	Tonal + time pressure	
Performance time (s)	6.6 (1.0)	6.4 (0.8)	4.9 (0.6)	7.6 (1.6)	6.6 (1.1)	5.0 (0.9)	C, G × C
Lead vertical toe clearance (m)	0.17 (0.03)	0.16 (0.02)	0.15 (0.03)	0.24 (0.05)	0.21 (0.04)	0.24 (0.06)	G, C, G × C
Trail vertical toe clearance (m)	0.16 (0.05)	0.15 (0.05)	0.13 (0.03)	0.18 (0.05)	0.16 (0.05)	0.18 (0.05)	C
Lead horizontal toe velocity (m/s)	3.4 (0.4)	3.5 (0.5)	4.6 (0.7)	3.0 (0.6)	3.2 (0.5)	4.1 (0.8)	G, C
Trail horizontal toe velocity (m/s)	2.8 (0.6)	3.0 (0.6)	3.9 (0.6)	2.9 (0.6)	3.0 (0.5)	4.4 (0.9)	C
Penultimate foot placement (m)	0.99 (0.18)	0.97 (0.15)	1.14 (0.20)	0.92 (0.27)	0.97 (0.17)	1.14 (0.18)	C
Final foot placement (m)	0.27 (0.06)	0.26 (0.06)	0.31 (0.09)	0.30 (0.09)	0.31 (0.08)	0.39 (0.09)	G, C
Single support time lead foot (s)	0.59 (0.04)	0.56 (0.03)	0.49 (0.05)	0.66 (0.08)	0.61 (0.07)	0.52 (0.06)	G, C
Single support time trail foot	0.51 (0.04)	0.50 (0.05)	0.45 (0.04)	0.50 (0.03)	0.50 (0.05)	0.45 (0.03)	C

G, significant group effect (*p* < 0.05); C, significant condition effect (*p* < 0.05); G × C, significant group by condition interaction (*p* < 0.05); n/a, no significant effect.

The order in which the conditions were presented did not affect the task performance times as performance times within each condition were not different for the participants who started with the no pressure vs. tonal + time pressure condition (all *p* > 0.05).

### Questionnaires

**Figure 3:**
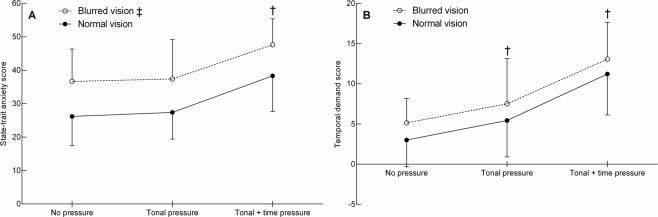
Data of the State-Trait Anxiety Inventory (**A**) and perceived temporal demand (**B**) for the normal vision and blurred vision group in the three different conditions (mean ± SD). ^‡^The score pooled across conditions differs significantly between groups (*p* < 0.05). ^†^The score pooled across groups differs significantly from the other conditions (*p* < 0.05).

Figure 3A shows the outcomes of the Spielberger State-Trait Anxiety Inventory. A group effect showed that the blurred vision group reported 32% more anxiety than the normal vision group (*F*_1,26_ = 10.0, *p* = 0.004, *d* = 1.20). A condition effect revealed that subjects perceived 33–37% more anxiety in the tonal + time pressure condition compared to the tonal pressure and no pressure conditions (*F*_2,52_ = 34.3, *p* < 0.001). There was no difference in anxiety between the tonal pressure and no pressure conditions (*p* = 1.00). No group by condition interaction was found (*p* = 0.928).

Figure 3B shows the temporal demand score of the NASA Task Load Index. No group effect was observed (*p* = 0.128). The condition effect revealed that the temporal demand score increased with increasing pressure (*F*_2,52_ = 25.2, *p* < 0.001). No group by condition interaction was observed (*p* = 0.988).

### Movement kinematics

Movement kinematics were collected at 100 Hz using a six camera 3-D motion capture system. Reflective markers were placed on the left and right shoe and the upper front edge of the obstacle to determine the height and location of the obstacle within the laboratory coordinate system.

#### Vertical toe clearance height

**Figure 4:**
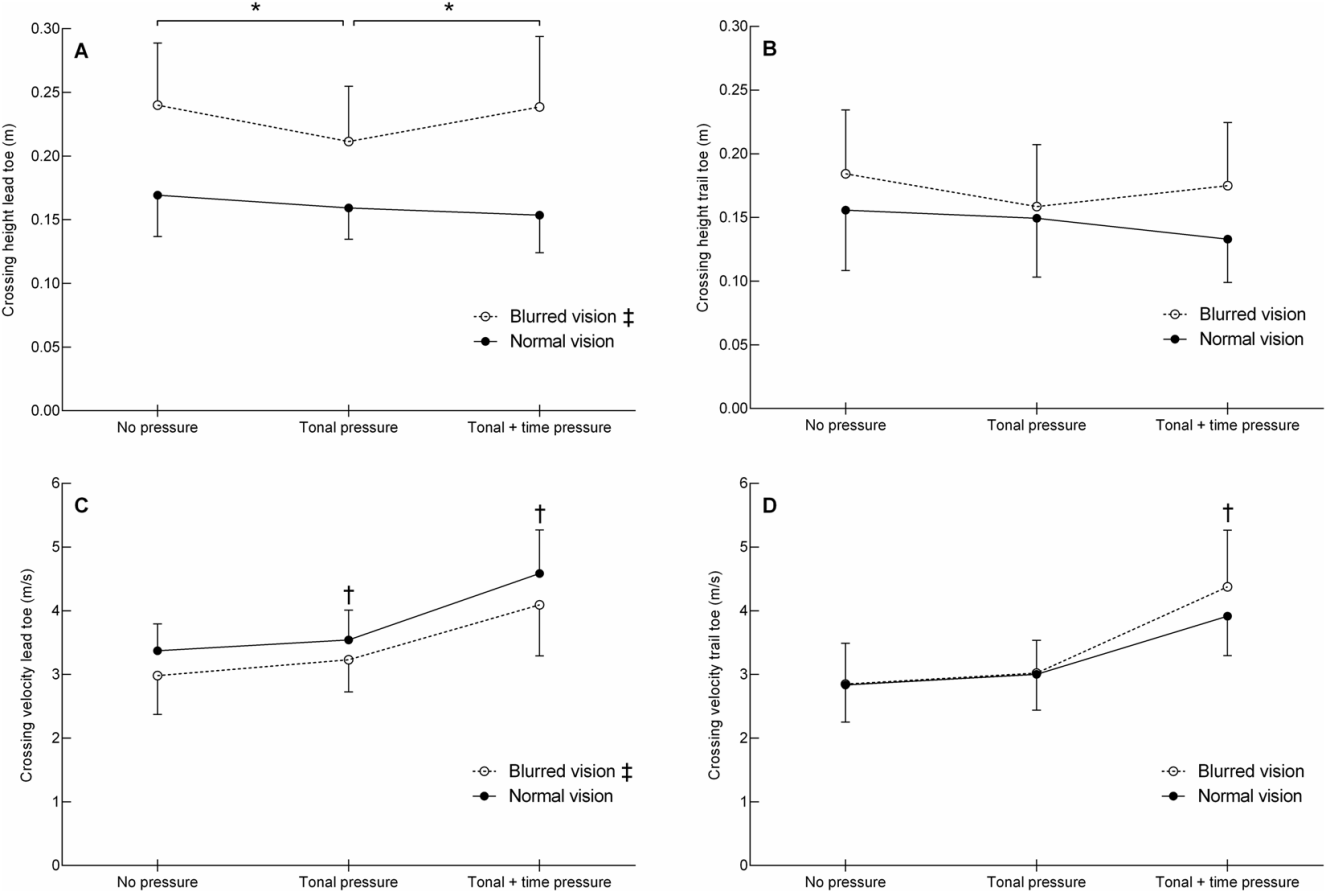
Clearance heights (**A**, lead toe; **B**, trail toe) and horizontal crossing velocities (**C**, lead toe; **D**, trail toe) for the normal vision and blurred vision group in the three different conditions (mean ± SD). ^‡^The score pooled across conditions differs significantly between groups (*p* < 0.05). ^†^The score pooled across groups differs significantly from the other two conditions (*p* < 0.05). *Group by condition interaction (*p* < 0.05).

The vertical toe clearance height was calculated as the vertical distance between the toe maker and the obstacle at the instant of obstacle crossing. Figure 4 (panels A,B) and Table 2 show the vertical toe clearance heights for the lead and trail foot. The clearance height of the lead toe showed a group effect (*F*_1,26_ = 25.8, *p* < 0.001), condition effect (*F*_1,36_ = 5.1, *p* = 0.02), and group by condition interaction (*F*_1,36_ = 3.7, *p* = 0.049). Post hoc testing for the interaction effect revealed that the clearance height was 43% higher for the blurred vision group than normal vision group in all three conditions (all *p* ≤ 0.001). On comparing between conditions, the blurred vision group had a lower clearance height in the tonal pressure condition vs. the tonal + time pressure and no pressure condition (both *p* ≤ 0.002) whereas crossing height did not differ across conditions for the normal vision group (all *p* ≥ 0.106).

The trail toe showed no main group effect (*p* = 0.097) but a condition effect was found (*F*_2,52_ = 3.5, *p* = 0.039). However, Post hoc testing revealed no significant difference in clearance height between conditions (*p* ≥ 0.076). No group by condition interaction was observed (*p* = 0.074).

#### Horizontal crossing velocity

The horizontal crossing velocity was calculated from the toe marker at the instant of obstacle crossing. Figure 4 (panels C,D) and Table 2 show the horizontal crossing velocity of the lead and trail foot. There was a significant group effect for the lead toe (*F*_1,26_ = 4.8, *p* = 0.037, *d* = 0.81) showing that the crossing velocity was 10% slower for the blurred vision than normal vision group. There was also a main effect of condition (*F*_2,41_ = 57.1, *p* < 0.001) showing that the crossing velocity of the lead toe increased with increasing pressure (all *p* ≤ 0.042). No group by condition interaction was found (*p* = 0.683).

The crossing velocity of the trail toe did not show a group effect (*p* = 0.425) but there was a significant main condition effect (*F*_2,52_ = 74.8, *p* < 0.001). Post hoc testing showed that the crossing velocity of the trail toe was 38% higher in the tonal + time pressure vs. tonal pressure condition (*p* < 0.001, *d* = 1.65), and 46% higher in the tonal + time pressure vs. no pressure condition (*p* < 0.001, *d* = 1.84). No group by condition interactions was found (*p* = 0.098).

#### Foot placement

Foot placement was calculated as the horizontal distance between the toe marker and the obstacle at 0.01 s (1 frame) before toe off (i.e., the instant where the resultant velocity of the foot’s toe maker first increased more than 0.9 m/s for ten consecutive frames). The results for penultimate and final foot placement can be found in Table 2. Penultimate foot placement did not show a group effect (*p* = 0.742) but a main condition effects was observed (*F*_2,52_ = 20.6, *p* < 0.001). Post hoc testing of the condition effect revealed that subjects placed their foot further away in the tonal + time pressure condition vs. the tonal pressure (*p* < 0.001, *d* = 0.97) and no pressure condition (*p* < 0.001, *d* = 0.85). No group by condition interaction was observed (*p* = 0.493).

A group effect for final foot placement was observed showing that the blurred vision group compared to the normal vision group placed their final foot 20% further away from the obstacle before crossing (*F*_1,26_ = 4.3, *p* = 0.048, *d* = 0.77). The condition effect (*F*_2,52_ = 16.0, *p* < 0.001) revealed that compared to the tonal + time pressure condition, subjects placed the final foot closer to the obstacle in the tonal pressure (*p* < 0.001, *d* = 0.77) and no pressure condition (*p* < 0.001, *d* = 0.77). No group by condition interaction was observed (*p* = 0.334).

#### Single support time

Table 2 contains single support times of the lead and trail limb during crossing. The single support time of the lead limb is defined as the swing time of the lead limb during obstacle crossing whereby only the trail limb is in contact with the ground (the trail limb has not crossed the obstacle yet). The single support time of the trail limb is defined as the swing time of the trail limb during obstacle crossing whereby only the lead limb is in contact with the ground (the lead limb has already crossed the obstacle). The group effect for the single support times of the lead limb is showing that single support is 9% longer in the blurred vision than normal vision group (*F*_1,26_ = 7.5, *p* = 0.011, *d* = 1.00). A condition effect was also observed (*F*_2,52_ = 52.8, *p* < 0.001). Post hoc testing of the condition effect revealed that single support times become shorter with increasing pressure (all *p* ≤ 0.004). No group by condition interaction was observed (*p* = 0.258).

The single support times of the trail limb during obstacle crossing showed no group effect (*p* = 0.795) but a main condition effect was observed (*F*_2,52_ = 34.5, *p* < 0.001). Post hoc analysis of the condition effect revealed that single support times became shorter in the tonal + time pressure condition compared to the tonal pressure (*p* < 0.001, *d* = 1.10) and no pressure condition (*p* < 0.001, *d* = 1.50). No interaction effect was found (*p* = 0.598).

### Gaze behaviour

**Figure 5:**
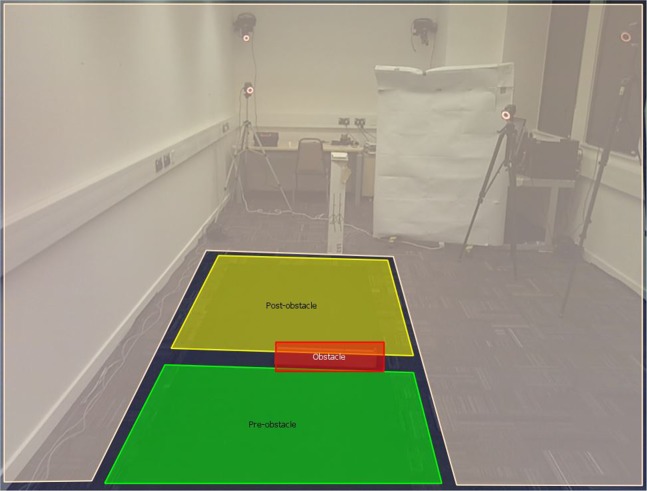
The areas of interest overlaid for tracking gaze behaviour.

Gaze behaviour was recorded in 27 participants; calibration of the eye tracker failed in one participant of the blurred vision group. Gaze behaviour was collected at 30 Hz using a head mounted mobile eye tracker. Eye tracker data were subjected to a manual frame-by-frame analysis using the following areas of interest (see also Fig. 5):

1. Pre-obstacle – gaze is down on the walkway prior to the obstacle.
2. Obstacle – gaze is on the obstacle.
3. Post-obstacle – gaze is on a section of the walkway that is ahead of the obstacle.

The following variables were obtained from the output of the manual mapping for each trial:

1. Trial length (s) – first frame of the flashing LED above the start pad (denoting the trial start) up to the frame where the hand touched the end pad (denoting the end of the trial).
2. Fixations – total number of gaze changes.
3. Scan rate (number/second) – total number of gaze changes divided by the total trial length.
4. Relative number of fixations (% of the total number of fixations) – total number of fixations at an area of interest as a percentage of the total number of gaze changes within a trial.
5. Relative fixation duration (% of total trial time) – total duration that a participant was looking at an area of interest as a percentage of the total trial length.

Table 3 shows the data of all gaze behaviour variables. A group effect was observed for the relative number of fixations post-obstacle (*F*_1,25_ = 11.5, *p* = 0.002, *d* = 0.85) and the relative fixation duration post-obstacle (*F*_1,25_ = 6.6, *p* = 0.017, *d* = 0.69). Post hoc analysis of the group effects revealed that the blurred vs. normal vision group fixated more often (24 vs. 18%) and for longer (19 vs. 13%) at the post-obstacle region. These changes in visual search are likely at the expense of the blurred vision group looking at the pre-obstacle region less frequently (−5%, *p* = 0.122) and for a shorter period of time (−4%, *p* = 0.100).

**Table 3:** Gaze behaviour of the normal vision and blurred vision group in the three different conditions (mean ± SD).

Variables	Normal vision group (n = 14)			Blurred vision group (n = 13)			ANOVA
	No pressure	Tonal pressure	Tonal + time pressure	No pressure	Tonal pressure	Tonal + time pressure	
Total number of fixations	16 (2)	15 (2)	13 (4)	16 (5)	15 (4)	14 (4)	C
Scan rate (number/s)	3 (1)	3 (1)	3 (1)	2 (1)	2 (1)	3 (2)	n/a
Number of fixations (% of total)							
At obstacle	17 (8)	16 (8)	17 (8)	19 (10)	20 (8)	15 (9)	n/a
At pre-obstacle	13 (8)	17 (10)	17 (10)	11 (7)	11 (9)	11 (10)	n/a
At post-obstacle	20 (7)	16 (4)	18 (7)	25 (9)	14 (8)	23 (7)	G
Fixation duration (% of total)							
At obstacle	11 (5)	12 (6)	9 (6)	14 (10)	14 (8)	8 (7)	C
At pre-obstacle	9 (7)	11 (8)	10 (8)	5 (4)	6 (7)	7 (8)	n/a
At post-obstacle	14 (5)	12 (5)	14 (6)	21 (11)	17 (7)	18 (9)	G

G, significant group effect (*p* < 0.05); C, significant condition effect (*p* < 0.05); n/a, no significant effect.

A condition effect was found for the total number of fixations (*F*_1,50_ = 7.6, *p* = 0.001), and the relative fixation duration at the obstacle (*F*_1,50_ = 4.1, *p* = 0.021). Post hoc analysis of the condition effects revealed that the total number of fixations was lower in the tonal + time pressure vs. no pressure condition (all *p* < 0.05). The relative fixation duration at the obstacle was shorter in the tonal + time pressure condition compared to the tonal pressure and no pressure conditions (all *p* < 0.05). The decrease in relative fixation duration was not accompanied by a specific increase for any of the other areas of interest (all *p* ≥ 0.237). No group by condition interactions were observed.

## Discussion

The present study investigated the effects of time-pressure and vision loss on the movement kinematics and gaze behaviour during an obstacle crossing task. Participants were randomly assigned to a normal vision or blurred vision group, and performed the task under no pressure, tonal pressure, and tonal + time pressure conditions. Key findings suggest that irrespective of the task demands, the blurred compared to normal vision group were more anxious, lifted the lead foot more cautiously over the obstacle (increased vertical height and reduced horizontal crossing velocity), and looked for longer and more frequently at the area ahead of the obstacle.

Anxiety levels were higher in the blurred vision than normal vision group, a finding similarly reported in older adults with non-simulated vision impairment^40,41^. The higher levels of perceived anxiety in the blurred vision group were also accompanied by, higher and slower obstacle crossing with the lead leg resulting in a longer single support phase, final foot placement further away from the obstacle, and slower task performance in the no pressure condition. A reduction in walking speed in temporally unconstraint situations has been shown before in individuals with blurred vision^8,10,11,13^ and could be a strategy to obtain more task-relevant information that allows better visuomotor planning^8^. A second adaptation to avoid obstacle contact is to lift the feet higher and slower over the obstacle^47^ as illustrated by the blurred vision group in the present study. Interestingly and similar to a study in cataract patients^9^, only the lead foot was lifted higher and slower over the obstacle, possibly because the crossing trajectory of the lead foot is modified online where the trajectory of the trail foot relies primarily on feedforward visual information^48,49^. The online control of the lead foot relies on exproprioceptive information^2^ that could lack detail when vision is blurred. Therefore, similar to when the peripheral visual field is blocked^2^, final foot placement was further away from the obstacle followed by an increase in toe clearance of the lead toe. The blurred visual information obtained in the approach phase was sufficient to guide the trail leg trajectory as the trajectory was not modified compared to the normal vision group. A disadvantage of the slower and higher crossing trajectory of the lead foot is the longer single support phase during crossing, challenging the dynamic stability, which is worse in individuals with blurred vision compared to those with normal vision^50^.

Irrespective of group, perceived anxiety was highest and the performance times were fastest in the tonal + time pressure condition. No differences for these two variables were found between the tonal pressure and no pressure condition. The tonal pressure condition was included to confirm that it was not the sound of the tone itself but the time-pressure component was the cause of the increase in anxiety and walking speed. In contrast to our hypothesis, higher levels of anxiety did not result in adaptations in movement kinematics that increased the risk of tripping on the obstacle. Our hypothesis was based on research where the travel path contained more than one floor based hazard^26,28,36^ thus it might be that anxiety-induced changes in obstacle crossing behaviour only become apparent when the walking task is more challenging. A more challenging task with multiple obstacles would have increased the cognitive load of the task which, together with a more conscious control of locomotion due to anxiety, could have evoked changes in awareness^30,35^ and obstacle negotiation that increased the risk of tripping. The kinematic changes observed in the present study were associated with walking faster due to the increased temporal demand in the tonal + time pressure vs. no pressure condition. To illustrate, foot placement was 19–22% further away from the obstacle before crossing, the horizontal crossing velocity of the lead and trail toe increased respectively 37% and 46%, and the time spent in single-limb support decreased 12–19%. The crossing height of the lead and trail toe were not altered by faster walking speeds. To date, one study has investigated the effects of walking speed on adaptive gait and reported data only for the trail foot^51^. Their data support our findings because with increasing speed, the trail foot was placed further away from the obstacle without affecting the crossing height^51^. Altogether, alterations in adaptive gait in a time constraint situation seem to be driven by walking speed and not anxiety.

In contrast to our hypothesis, we did not show significant alterations in adaptive gait due to the increased temporal demand between the blurred vs. normal vision group. It might be that the amount of vision loss has to be more severe before it will affect the visuomotor planning in time constraint situations^52^. On the other hand, it was reasonable to expect that simulated vision loss would produce more severe changes to gait and gaze behaviour. However our study does not show this, and it is possible that more severe visual impairment is required, although the level at which we simulated vision loss was relatively high; meeting the minimum threshold to be classified as visually impaired. Perhaps it needs to be higher still and examples in sports practice show that levels of performance start only to deteriorate when vision was blurred to plus 10 dioptres^53,54^. These studies did not measure performance in a temporally constraint situation. Future studies are required to examine whether higher amounts of vision loss can alter adaptive gait in time constraint situations.

The present study is the first to examine visual search behaviour in individuals with blurred vision when negotiating an obstacle. The visual search strategy was not different between the blurred and normal vision group except that the blurred vision group looked 6% longer and 6% more often ahead of the obstacle. These changes in visual search were likely at the expense of looking less often at the pre-obstacle region (−5%, *p* = 0.122) and reducing the time spent looking at the pre-obstacle region (−4%, *p* = 0.100). The post-obstacle area of interest is large in extent so it remains uncertain whether the blurred vision group looked longer and more frequently ahead to guide foot placement (i.e., looking at more immediate areas after the obstacle) or to scan the future travel path. This needs to be explored in future studies. In addition, the blurred vision group did not have to adapt their visual search strategy to accurately detect the obstacle. This finding agrees with previous research which showed that the detection of 3-D shapes is relatively well preserved when visual acuity was reduced to 0.92 logMAR due to blur^55^.

Irrespective of group, the visual search behaviour was altered with increasing temporal demand. Individuals adopted a ‘checking strategy’ in the tonal + time pressure vs. no pressure condition by reducing the duration but not the frequency of looking at the obstacle. One can understand that in a temporally constrained environment, with less time available to look at features, the viewing duration is reduced to allow all key areas in the travel path to be checked. This strategy permits sufficient visual information in a shorter time to be perceived, to avoid tripping and falling on hazards that were otherwise not seen.

Young adults volunteered in the present study. It is known that the prevalence of vision loss is higher in adults aged 50 + years^56^. However it was important, in the first study of its kind, to ensure that reduced mobility due to age did not contribute to any effects observed. Future work in this area should consider testing older adults with actual visual impairment.

The magnification effect of positive trial lenses may have affected adaptive gait in the blurred vision group^57^. Objects appear closer with increased magnification and consequently the final foot is placed further away from the step and the crossing height of the lead foot but not the trail foot is increased when negotiating a step^57^. Similar results for these variables were found in the present study and could be attributed to magnification. However, it is unlikely that the group effects in the present study are the result of magnification only as the magnification effect is about 0.5 cm/dioptre for lead toe clearance^57^ while lead toe clearance in the present study was 7 cm higher when participants were blurred with trial lenses of plus 4–6 dioptres.

In conclusion, this is the first study to examine the effects of time-pressure during an everyday walking task in individuals with and without blurred vision. Irrespective of temporal demand, the blurred vs. normal vision group were more anxious and negotiated the obstacle more cautiously with the lead foot, to reduce the likelihood of tripping on the obstacle. The blurred vision group also looked longer and more frequently ahead of the obstacle to facilitate safe foot placement or to scan the future travel path. Performing the task under time-pressure resulted in changes in crossing behaviour that were associated with walking faster. The increased temporal demand also changed visual search behaviour, resulting in participants adopting a ‘checking strategy’ to obtain sufficient visual information of the environment with less time available to complete the task. Future research should address the effects of time-pressure on the performance of everyday walking tasks in individuals with habitual/chronic vision impairment. Knowing how individuals with vision loss perform everyday tasks will help in the design of interventions that improve adaptive gait and gaze behaviour, in an attempt to decrease their risk of falling.

## Methods

### Participants

A total of 28 healthy volunteers with normal or corrected-to-normal vision were recruited for the study. Participants were randomly assigned to a normal vision group (*n* = 14) and blurred vision group (*n* = 14). The group characteristics can be found in Table 1. Visual acuity in the blurred vision group was impaired using full aperture trial case lenses of plus 4 to plus 6 dioptres (Wholesale Lens Co Ltd, Croydon, United Kingdom), to ensure all participants met the minimum threshold to be classified as visually impaired (according to their visual acuity score (0.95 ± 0.07 logMAR)) resembling sight impaired individuals (partially sighted) according to the Certificate of Vision Impairment in England and Wales^58^. Self-reported auditory function was normal in all participants. All participants provided written informed consent to the experimental procedures, which were approved by the Research Ethics Committee of the Anglia Ruskin University and in accordance with the Declaration of Helsinki.

### Experimental setup

Figure 1 shows a schematic representation of the experimental setup. The walkway was 7 m in length, 1.2 m wide and positioned in the middle of a research laboratory. Participants started behind a cardboard wall that blocked the participants’ view of the travel path at the start of each trial. Before every trial, participants were instructed to place the hand on a pressure pad and look at an LED light at the top of the pressure pad. Every trial started after the sound of a single ‘beep’. Then, participants released their hand from the pressure pad, turned 90 degrees in clockwise direction and started walking along the travel path. The trial was completed when the participants placed their hand on the second pressure pad at the end of the walkway. The cardboard wall at the end of the walkway prevented the participants from seeing the researchers. The trials were timed using pressure sensors underneath the pad. Timing started when the hand was released from the ‘start pad’ and stopped when the hand was placed at the ‘end pad’. The height of the pad stand was 88 cm for the pressure pad at the start and 92 cm for the pressure pad at the end. In the obstacle crossing trials, the obstacle was randomly positioned between 4.05 and 5.30 m from the start position so the participants had to adjust their gait between trials and avoided any learning effect through adopting a repeated motor pattern. The height of the obstacle was 10 cm and reflected a typical height encountered in everyday life^59^. The obstacle was 1.8 cm thick and 62 cm long and constructed from light brown medium-density fibreboard. The colour of the obstacle contrasted with the black background of the laboratory carpet.

The participants in the blurred vision group were allowed to observe the laboratory with their full visual capacity before they were blurred. The experiment started immediately after the blur was evoked using the trial lenses, leaving minimal time to get accustomed to the lenses. Participants started the experiment with nine baseline normal walking trials (no obstacle present) at their comfortable walking speed (i.e., to get familiar with the task and walking surface) followed by trials where they had to negotiate a floor-based obstacle under tonal + time pressure, tonal pressure, and no pressure conditions. Tonal + time pressure was induced by a custom-made timing device that produced an intermittent tone that increased in frequency. The intermittent tone with a loudness of approximately 82 dB started once the participant released the hand from the ‘start pad’ and stopped when the hand was placed at the ‘end pad’. This tone required the subjects to complete the task 20% faster than their comfortable walking speed. Participants’ comfortable walking speed was calculated as the average walking speed over the last three baseline normal walking trials. Participants were instructed to reach the ‘end pad’ before the tone extinguished without running. Participants heard the intermittent tone once (without performing the obstacle crossing task) before they commenced the first trial of the tonal + time pressure block. In the tonal pressure condition, an intermittent tone at a constant frequency was played. This condition was added to confirm that the effects in the tonal + time-pressure condition were evoked by the temporal component and not by the tonal component of the task as a tone itself can cause stress^60^ and impair motor performance^17^. In the no pressure condition, no tone was played and participants were instructed to walk at their comfortable walking speed. Every condition consisted of four trials of which three trials were with the obstacle and one trial without the obstacle (i.e. level walking trial). Before every trial, participants were unaware whether there was an obstacle present or not. Participants were aware of the condition they would face and practice trials were not allowed for any condition. Subjects randomly started with the no pressure or tonal + time pressure condition and always finished with the tonal pressure condition.

### Questionnaires

Anxiety and perceived temporal demand were examined after every condition (i.e. after finishing the block of trials in that condition). Anxiety was assessed using the short version of the Spielberger State-Trait Anxiety Inventory^61^. This questionnaire is reliable (Cronbach’s α is 0.82), has good concurrent validity and is sensitive to change^61^. The short version consisted of six items (I feel calm; I feel tense; I feel upset; I am relaxed; I am content: I am worried) and responses were scored on a Likert scale (1 = not at all to 4 = very much). The scores on the positive items (i.e., calm; relaxed; content) were reversed before summation of all scores. The total score was multiplied by 20/6 to give the final anxiety score. The final score ranged from 20–80 and a score between 34–36 is deemed ‘normal’^62^.

Perceived temporal demand was examined using the NASA Task Load Index^63^. The NASA Task Load Index is comprised of six items that assess mental, physical, and temporal demand (“How hurried or rushed was the pace of the task”), and perceived performance, effort, and frustration. Participants rate five items on a bipolar scale that ranges from 0 = very low to 20 = very high and one item (i.e., performance) on a bipolar scale that ranges from 0 = perfect to 20 = failure. Normally, the total perceived workload is calculated by taking the average score of the six items^64^ but in the present study only the temporal demand score was analysed.

### Movement kinematics

Movement kinematics were collected at 100 Hz using a six camera 3-D motion capture system (Vicon, Oxford Metrics Ltd). Reflective markers were placed on the left and right shoe (toe, fifth proximal phalanx, medial and lateral side of the posterior part of the calcaneus), left and right hand (nail of the thumb, nail of the index finger, processus styloideus), upper front edge of the obstacle to determine the height and location of the obstacle within the laboratory coordinate system (two markers), and the edges of the end pad (four markers) to determine when the hand touched the pad (denoting the end of a trial). The kinematic data were filtered with a fourth-order low-pass Butterworth filter at 7 Hz. The gait analysis was performed using Visual 3D (C-Motion Inc., Rockville, MD., USA).

Heel strike with the floor during walking was defined as the instant where the resultant velocity of the foot’s medial heel marker first reduced less than 0.6 m/s for ten consecutive frames. Toe-off was defined as the instant where the resultant velocity of the foot’s toe maker first increased more than 0.9 m/s for ten consecutive frames. Both threshold values were determined by visual inspection of the kinematic data.

The following kinematic variables were examined, which have previously been identified as important in the assessment of adaptive gait^2,59,65^:

1. Vertical clearance height of the toe at the point of crossing the obstacle.
2. Horizontal crossing velocity of the toe at the point of obstacle crossing.
3. Penultimate and final foot placement before crossing the obstacle – horizontal distance between the toe and the obstacle.
4. Lead limb single support time – swing time of the lead limb during obstacle crossing whereby only the trail limb is in contact with the ground.
5. Trail limb single support time – swing time of the trail limb during obstacle crossing whereby only the lead limb is in contact with the ground.

### Gaze behaviour

Gaze behaviour was recorded in 27 participants; calibration of the eye tracker failed in one participant of the blurred vision group. Gaze behaviour was collected at 30 Hz using an SMI iView ETG head mounted mobile eye tracker (version 1.0; SensoMotoric Instruments, Teltow, Germany). Additional information about the eye-tracker system has been published previously^66^. The lenses (used in the blurred group only) were attached to the front, outside of the eye tracker such that it would not influence the eye tracker’s ability to track eye movements. A three-point eye calibration was performed to verify the participants’ point-of-gaze. Three key features in the laboratory were used as target points for the calibration. The calibration was checked after every fourth trial. Data were recorded on a mini laptop (Lenovo X220; ThinkPad, Boston, MA, USA) with SMI iView ETG recording software installed on it (version 2.0; SensoMotoric Instruments). The laptop was carried in a backpack by the participant.

Eye tracker data was analysed offline using SMI BeGaze software (version 3.4; SensoMotoric Instruments) and was subjected to frame-by-frame analysis. Each trial was tracked from the frame that the LED above the start pad started flashing (i.e., start of the trial) up to the frame where the hand made contact with the end pad (i.e., end of the trial). The areas of interest were adopted from a previous obstacle crossing study^66^. The following areas of interest were used (see also Fig. 5):

1. Pre-obstacle – gaze is down on the walkway prior to the obstacle.
2. Obstacle – gaze is on the obstacle.
3. Post-obstacle – gaze is on a section of the walkway that is ahead of the obstacle.

Each point-of-gaze was mapped manually to one of the areas of interest. A coding window was created to ensure that every change in gaze within and between areas of interest was recorded (i.e., number of fixations during a trial). This coding window made it possible to record changes in gaze within the same area of interest^66^. The output of the manual mapping was analysed using a custom Matlab script (version 2016a; The Mathworks, Natick, Mass., USA). The following variables were determined for each trial:

1. Trial length (s) – first frame of the flashing LED above the start pad (denoting the trial start) up to the frame where the hand touched the end pad (denoting the end of the trial).
2. Fixations – total number of gaze changes.
3. Scan rate (number/second) – total number of gaze changes divided by the total trial length.
4. Relative number of fixations (% of the total number of fixations) – total number of fixations at an area of interest as a percentage of the total number of gaze changes within a trial.
5. Relative fixation duration (% of total trial time) – total duration that a participant was looking at an area of interest as a percentage of the total trial length.

Previous work showed that the intra-rater and inter-rater reliability was excellent for this eye-tracker when using a similar approach for the data analysis as in the present study^66^. To illustrate, the intra-rater reliability agreement was 95% on average for all variables and the inter-rater reliability agreement was 94% on average for all variables^66^. The tracking ratio of the eye-tracker data in all participants was above 90%, deemed to be acceptable for analysis^67^.

### Simultaneous recording of movement kinematics and gaze behaviour

Movement kinematics and gaze behaviour were simultaneously recorded using a custom Python script (version 2.7; Python Software Foundation). This code simultaneously resulted in a single ‘beep’ (i.e., trial start), initiation of the data collection of the 3-D movement kinematics, and the flashing of an LED positioned on top of the start pad. The flashing of the LED was recorded by the eye tracker and provided the start point for the data analysis of gaze behaviour.

### Statistical analysis

Data in text and figures are expressed as mean ± SD. The statistical analysis was performed using SPSS version 23. Each variable was checked for normality. All variables were analysed using a group (normal vision, blurred vision) by condition (no pressure, tonal pressure, tonal + time pressure) mixed ANOVA to determine between- and within-subject effects. Significant *F* values from the ANOVA’s were subjected to a Bonferroni post hoc pairwise comparison to determine the means that were different. The level of significance (α) was set at *p* < 0.05. Effect sizes were calculated using Cohen’s *d*. The sample size was based on a previous study that reported differences in movement kinematics between individuals with vs. without central field loss when negotiating a low and high obstacle^59^.

## Data Availability

The data that support the findings of this study are available from the corresponding author, T.Z., upon reasonable request.
